# Detection of HTLV-1 proviral DNA in cell-free DNA: Potential for non-invasive monitoring of Adult T cell leukaemia/lymphoma using liquid biopsy?

**DOI:** 10.3389/fimmu.2023.1150285

**Published:** 2023-04-11

**Authors:** Thomas Joris, Jana Haddow, Graham P. Taylor, Lucy B. M. Cook, Aileen G. Rowan

**Affiliations:** ^1^ Section of Virology, Department of Infectious Disease, Imperial College London, London, United Kingdom; ^2^ Gembloux Agro-Biotech, University of Liege, Gembloux, Belgium; ^3^ National Centre for Human Retrovirology, Imperial College Healthcare National Health Service Trust, London, United Kingdom; ^4^ Department of Haematology, Imperial College Healthcare National Health Service (NHS) Trust, London, United Kingdom; ^5^ Centre for Haematology, Department of Immunology and Inflammation, Imperial College London, London, United Kingdom

**Keywords:** cell-free DNA, liquid biopsy, HTLV-1, adult T cell leukaemia/lymphoma, provirus, retrovirus

## Abstract

**Introduction:**

Fragmented genomic DNA is constitutively released from dying cells into interstitial fluid in healthy tissue. In cancer, this so-called ‘cell-free’ DNA (cfDNA) released from dying malignant cells encodes cancer-associated mutations. Thus, minimally invasive sampling of cfDNA in blood plasma can be used to diagnose, characterise and longitudinally monitor solid tumours at remote sites in the body. ~5% of carriers of Human T cell leukaemia virus type 1 (HTLV-1) develop Adult T cell leukaemia/lymphoma (ATL), and a similar percentage develop an inflammatory CNS disease, HTLV-1 associated myelopathy (HAM). In both ATL and HAM, high frequencies of HTLV-1 infected cells are present in the affected tissue: each carrying an integrated DNA copy of the provirus. We hypothesised that turnover of infected cells results in the release of HTLV-1 proviruses in cfDNA, and that analysis of cfDNA from infected cells in HTLV-1 carriers might contain clinically useful information pertaining to inaccessible sites in the body- e.g. for early detection of primary or relapsing localised lymphoma type ATL. To evaluate the feasibility of this approach, we tested for HTLV-1 proviruses in blood plasma cfDNA.

**Methods:**

CfDNA (from blood plasma) and genomic DNA (gDNA, from peripheral blood mononuclear cells, PBMC) was isolated from blood from 6 uninfected controls, 24 asymptomatic carriers (AC), 21 patients with HAM and 25 patients with ATL. Proviral (HTLV-1 *Tax*) and human genomic DNA (the beta globin gene, *HBB*) targets were quantified by qPCR using primer pairs optimised for fragmented DNA.

**Results:**

Pure, high quality cfDNA was successfully extracted from blood plasma of all study participants. When compared with uninfected controls, HTLV-1 carriers had higher concentrations of cfDNA circulating in their blood plasma. Patients with ATL who were not in remission had the highest levels of blood plasma cfDNA in any group studied. HTLV-1 proviral DNA was detected in 60/70 samples obtained from HTLV-1 carriers. The proviral load (percentage of cells carrying proviruses) was approximately tenfold lower in plasma cfDNA than in PBMC genomic DNA, and there was a strong correlation between the proviral load in cfDNA and PBMC genomic DNA in HTLV-1 carriers that did not have ATL. cfDNA samples in which proviruses were undetectable also had very low proviral load in PBMC genomic DNA. Finally, detection of proviruses in cfDNA of patients with ATL was predictive of clinical status: patients with evolving disease had higher than expected total amount of proviruses detectable in plasma cfDNA.

**Discussion:**

We demonstrated that (1) HTLV-1 infection is associated with increased levels of blood plasma cfDNA, (2) proviral DNA is released into blood plasma cfDNA in HTLV-1 carriers and (3) proviral burden in cfDNA correlates with clinical status, raising the possibility of developing assays of cfDNA for clinical use in HTLV-1 carriers.

## 1 Introduction

Dying cells release fragmented DNA into the extracellular environment, where it can transiently enter the blood flow before being fully degraded (1). If derived from a malignant cell, these fragments of cfDNA include somatic mutations which drive cancer (2, 3). At present, to diagnose most solid cancers, clinicians must collect tissue from tumours, traditionally by performing surgical biopsies. Apart from being highly invasive, surgical biopsies are subject to sampling bias, providing genomic and immunohistochemical information from a single mass at a single time point: other clinically significant tumour variants may arise during treatment, or even exist at distinct sites which were not sampled at the time of the original biopsy (4). Analysis of cfDNA in liquid biopsies permits non-invasive ‘global’ monitoring of genomic information from the whole body and can be repeated at intervals during treatment with minimal discomfort. The scope of this approach is only limited by the number and frequency of tumour-associated variant sequences themselves: anything from whole exome sequencing or targeted sequencing of known mutational hotspots can be performed to study tumours through the analysis of cfDNA.

Human T cell leukaemia virus type 1 (HTLV-1) causes the aggressive malignancy Adult T cell leukaemia/lymphoma (ATL) which presents as solid (lymphomatous and cutaneous subtypes) and liquid tumours (acute and chronic subtypes). Integration of a DNA proviral copy of the HTLV-1 genome into the host cell DNA is thought to be the first oncogenic ‘hit’ in the transformation to ATL, and malignant cells in the lymph nodes (lymphoma subtype) blood (leukemic subtype) (5, 6) and skin (cutaneous type) (7) each carry a copy of the HTLV-1 proviral genome as well as hundreds of somatic mutations in the host cell genome (8, 9).

Evaluation of the proviral load of peripheral blood mononuclear cells (PBMC PVL, percentage of PBMCs carrying integrated proviruses), is used clinically to determine risk of developing HTLV-associated diseases. PBMC PVL varies between HTLV-1-carriers by >5 logs, but is remarkably stable within an individual (6, 10). HTLV-1 carriers with PVLs >4% of PBMCs have a 20% lifetime risk of developing ATL (11), whereas carriers with PVLs below this threshold rarely develop ATL. When monitored longitudinally, a significant increase in PBMC PVL within an individual can provide early evidence of the onset of malignancy (12), and assays of PBMC PVL are also used to confirm a diagnosis of ATL (13) by demonstrating a high frequency of HTLV-1 infected cells in the malignant tissue. However, the PVL of PBMC does not always reflect disease progression/remission, particularly in patients with lymphoma type ATL, who currently have to undergo diagnostic biopsies to access the appropriate treatment. Therefore, there is a clinical need to develop clinically informative, minimally invasive novel methods to diagnose and monitor lymphoma subtype ATL to complement our existing methods.

Though the PVL of PBMC is very stable within an individual and viral gene expression is rarely detectable in ex vivo samples, HTLV-1 infected T cells turnover more often than uninfected T cells *in vivo (* 14). We hypothesised that cfDNA containing HTLV-1 proviruses are released by dying HTLV-1-infected cells into the extracellular fluid during the natural course of cellular turnover in all HTLV-1 carriers, raising the possibility that assays of cfDNA could be used to provide information about the burden of HTLV-1 infection in other tissues.

In order to determine whether DNA from HTLV-1 infected cells is present in cfDNA, we established an assay which quantifies proviruses in cfDNA, and compared the proviral burden in plasma cfDNA in patients with all subtypes of ATL, HTLV-1-associated myelopathy (HAM) and asymptomatic carriers of HTLV-1 with a range of PBMC PVLs. We showed that HTLV-1 proviruses could be detected in all patient groups, and the highest levels were detected in patients with ATL who were not in remission, indicating that a significant amount of ATL-derived nucleic acids are released into the blood plasma.

## 2 Methods

### 2.1 Blood samples

Blood donors attended the National Centre for Human Retrovirology (Imperial College Healthcare NHS Trust, St Mary’s Hospital, London). Written informed consent was obtained and research was conducted under the governance of the Communicable Diseases Research Group Tissue Bank, approved by the UK National Research Ethics Service (09/H0606/106, 15/SC/0089, 20/SC/0226).

### 2.2 DNA isolation

Whole blood collected in EDTA tubes was centrifuged at 1900g for 10 min within four hours of venepuncture. Directly after centrifugation, plasma was transferred to 2 ml tubes and centrifuged at 16,800g for 10 min at 4°C. The plasma supernatant was collected and stored at -80°C until cfDNA was purified using the QIAamp MinElute CCF-DNA kit (Qiagen). PBMC were isolated from the same blood samples using density gradient centrifugation. PBMC gDNA was purified using the QIAamp DNA Mini Kit (Qiagen) following manufacturers instructions. Before proceeding to qPCR, DNA was analysed by Agilent tapestation (cfDNA screentape) and quantified using a qubit (high sensitivity dsDNA kit).

### 2.3 Quantitative PCR

In each sample, the number of copies of the HTLV-1 *Tax* gene and the human beta-globin gene *HBB* was quantified using PowerUp SYBR Green Master Mix (ThermoFisher) and the following primers sets: SK43_58/SK44, 5’- CCACCTGTCCAGAGCATCAGA-3’/5’-GAGCCGATAACGCGTCCATCG-3’ (HTLV *Tax*, 58bp product); BG84F/BG84R, 5’-GCAAGGTGAACGTGGATG-3’/5’-TAAGGGTGGGAAAATAGACC-3’ (Human *HBB*, 172 bp product); BG84F/BG84R_80: 5’-GCAAGGTGAACGTGGATG-3’/5’-GGTCTCCTTAAACCTGTCTTGT-3’(Human *HBB*, 80 bp product). Serially diluted genomic DNA extracted from the MT2 cell line (which carries 7 integrated copies of HTLV-1 and two copies of *HBB* per cell), were used to generate a standard curve. PVL was calculated with the following formula: PVL= (*Tax* copies/(*HBB* 80bp copies/2))*100. Results were plotted as copies of Tax/ml of plasma and the percentage of cells carrying HTLV-1 proviruses (PVL) in PBMC gDNA and plasma cfDNA.

### 2.4 Data analysis

Graphs were prepared and statistical analysis was performed using GraphPad Prism Version 9.4.0. When paired data was analysed, paired statistical tests were used e.g. when comparing methods of quantification. When comparing two sets of paired data the Wilcoxon matched-pairs signed rank test was used, when comparing more than two sets of paired data the Friedman test with Dunn’s multiple comparison test was used. For unpaired data (e.g. when comparing PVLs of samples from several groups of people), the Kruskal-Wallis test with Dunn’s multiple comparison test was used. Pearson correlation analysis was used to evaluate whether PVL gDNA PVL could be used to predict plasma cfDNA PVL.

## 3 Results

### 3.1 Samples and cfDNA quantity

We studied peripheral blood samples from 76 donors: n=6 uninfected (UI), n=24 asymptomatic carriers (AC), n=21 with HAM and n=25 with ATL (**Supplementary Table 1**). Patients with ATL were separated into two groups based on their status at the time the sample was obtained. Patients with ATL who were in remission were placed in ATL-R group (n=13). Patients with ATL that had evidence of significant disease activity were placed in the ‘Not in Remission’ group (ATL-NR, n=12). Patients were assigned to each group on the basis of laboratory and/or clinical assessment and is outlined in detail in **Supplementary Table 2**.

**Figure 1:**
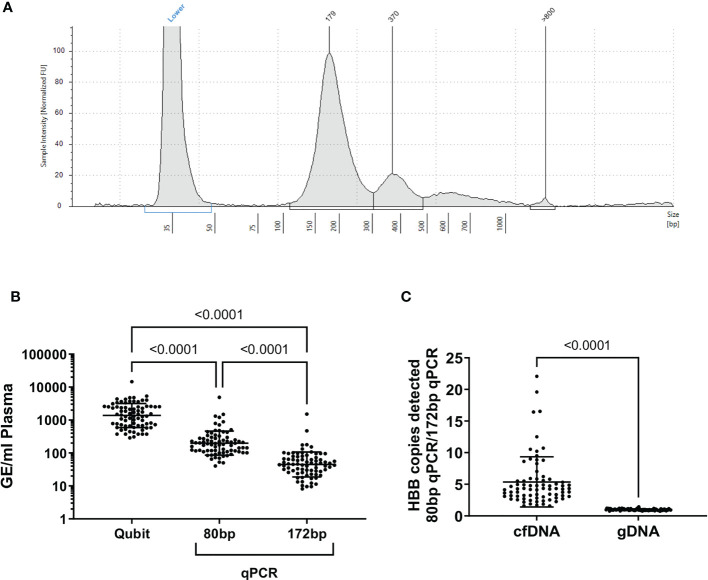
Quality assessment of cfDNA purified from plasma. **(A)** Representative electrophoretogram of purified plasma cfDNA (cfDNA screentape) **(B)** GE/ml of plasma calculated from each quantification method applied to plasma cfDNA. **(C)** Ratio of *HBB* copies detected with the 80bp *HBB* primers: 172 bp primers. In genomic DNA, there was no difference in the number of *HBB* copies detected with either primer (p=0.3, one sample t test). In cfDNA samples, significantly more *HBB* copies were detected with the 80 bp primer set than the 172 bp primer set, indicating that the cfDNA was highly fragmented. Statistical tests: **(B)** Friedman test with Dunn’s multiple comparisons test (two-tailed, 95% confidence interval), **(C)** Wilcoxon matched-pairs signed rank test (two-tailed, 95% confidence interval), using GraphPad Prism.

**Figure 2:**
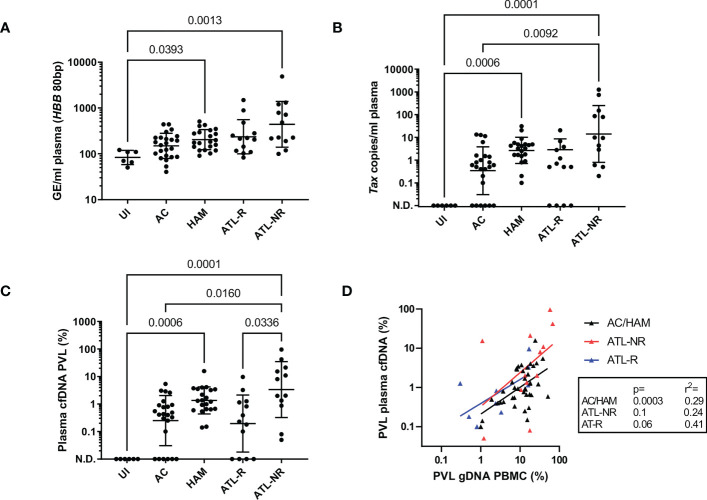
Detection of HTLV-1 in plasma cfDNA from HTLV-1 carriers. qPCR was used to quantify the copy number of Tax and *HBB* in plasma cfDNA and PBMC gDNA in blood donated by six uninfected controls, 24 ACs, 21 patients with HAM and 25 patients with ATL. Patients with ATL were either in remission (ATL-R, n=12) or were untreated, had stable disease or were being treated and had significant residual disease (Not in Remission, ATL-NR, n=13). **(A)** Genome equivalents (GE) per ml of plasma for each group were measured by quantifying the number of copies of *HBB* (80 bp PCR product). **(B)** Tax copies/ml of plasma (58bp PCR product). N.D. indicates not detected. **(C)** Plasma cfDNA PVL. PVL was calculated with the following formula: PVL= (*Tax* copies/GE(*HBB* 80bp))*100. **(D)** Simple linear regression of the PBMC genomic DNA (gDNA) PVL versus plasma cfDNA PVL in the AC/HAM, ATL-R and ATL-NR groups. Lines and error bars indicate the geometric mean and geometric SD. Statistical tests: Kruskal-Wallis with Dunn’s multiple comparisons test (two-tailed, 95% confidence interval, GraphPad Prism).

Cell-free DNA isolated from blood plasma was the expected size (~150bp, **Figure 1A**) (15), and there was no evidence of contamination with high molecular weight DNA in cfDNA samples (median cfDNA fraction >90% in all patients, cfDNA Tapestation). Plasma cfDNA was quantified by fluorometric assay (Qubit) and by two qPCR assays for the human beta globin gene (*HBB*) one which gave a 172bp product, and one which gave an 80bp product. By fluorometry, a median of 8.7 ng DNA (1459 genome equivalents, GE) was recovered from each ml of plasma. By qPCR, a median of 45 GE/ml plasma (172 bp product), and 187 GE/ml plasma (80 bp product) were detected (**Figure 1B**). The discrepancies in the number of GE detected are expected, as cfDNA is highly nicked and fragmented, and likely to amplify poorly, particularly if the target product length is greater than the average fragment size of the template. On average, the number of GE detected in plasma cfDNA using the 80bp product was 4 fold greater than the number of GE detected using the 172bp *HBB* qPCR assay (**Figure 1C**). In contrast, there was no significant difference in the number of GE detected in genomic DNA (gDNA) samples using either the 80bp or 172bp *HBB* qPCR assay. Interestingly, patients in the HAM (median 183 GE/ml HBB 80bp; range 91-509 GE/ml) and ATL-NR (227 GE/ml; 100-4880 GE/ml) groups had significantly more GE/ml of plasma than UI controls (90 GE/ml; 50-121 GE/ml) (**Figure 2A**).

### 3.2 Detection of proviral sequences in cfDNA

We designed a set of primers to detect the HTLV-1 *tax* gene which gave a 50bp PCR product, targeting a region of the HTLV-1 genome selectively retained in malignant cells in most ATL cases, despite frequent deletions in the 5’ portion of the proviral genome (16). We detected Tax DNA in 60/70 plasma cfDNA samples from HTLV-1-infected donors, and 0/6 uninfected controls (**Figure 2B**). All individuals with undetectable Tax in plasma cfDNA were in the AC or ATL-R groups. The number of copies of Tax detected was significantly higher in the ATL-NR group (median 9.6 copies/ml range 0.2-1256 copies/ml) versus ACs (0.5 copies/ml, undetectable-13.3 copies/ml). The median number of copies of Tax detected per ml of plasma was 3 in the HAM group, 0.5 in the ATL-R group and ranged from 0.1-31 copies/ml and 0-21 copies/ml respectively.

### 3.3 Comparison of proviral copy number in cfDNA and PBMC genomic DNA

We calculated the fraction of cfDNA which was derived from provirus-carrying cells and expressed it as PVL. The plasma cfDNA PVL was approximately tenfold less than the PBMC gDNA PVL in the AC, HAM, and ATL-R groups (**Figures 2C**, **S1A, B**), and correlated with the PBMC gDNA PVL (**Figure 2D**). Interestingly, there was no correlation between the PBMC gDNA PVL and the plasma cfDNA PVL in the ATL-NR group, with more proviral copies detected than expected in the cfDNA of ATL-NR (**Figure 2D**) when compared with other groups.

### 3.4 Anylate stability and longitudinal studies

**Figure 3:**
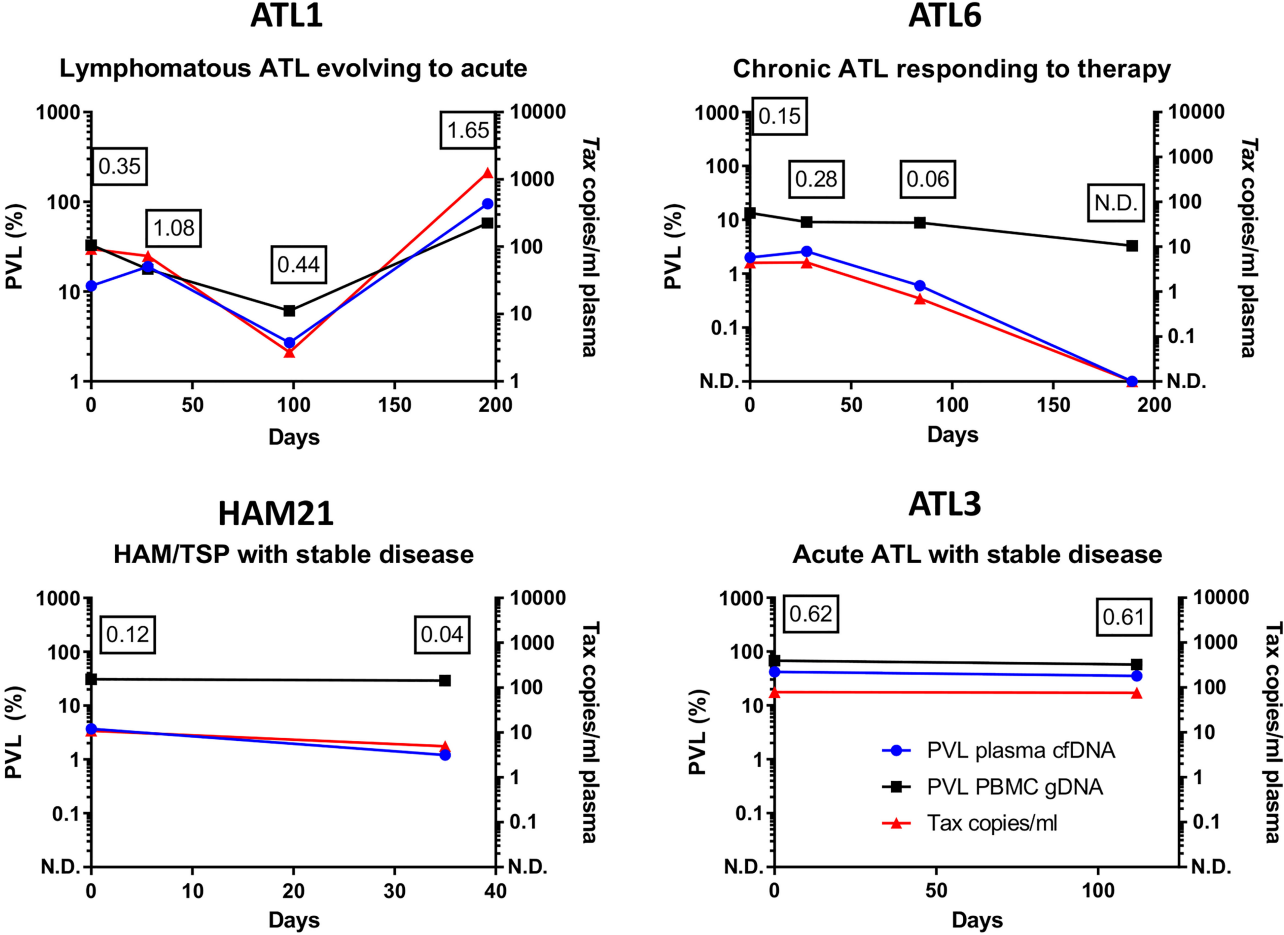
Longitudinal monitoring of HTLV-1 proviruses in cfDNA. qPCR was used to quantify the copy number of Tax and HBB in plasma cfDNA and PBMC gDNA in blood donated by four patients. Results were plotted as copies of Tax/ml and the percentage of cells carrying HTLV-1 proviruses (PVL) in PBMC gDNA and plasma cfDNA. Patient ATL1 had lymphomatous ATL which evolved to acute ATL on day 200. Patient ATL6 had chronic ATL was treated with Zidovudine/Interferon alpha (day 0-day 189 inclusive) and responded to therapy. Patients HAM21 (HAM) and ATL3 (Acute ATL) had stable disease. The ratio of the PVL of cfDNA to the PVL of PBMC gDNA is shown at each timepoint tested to illustrate differences between patients. N.D. indicates HTLV-1 Tax was not detected. Figures in boxes indicate the ratio of the plasma cfDNA PVL to the PBMC gDNA PVL at each timepoint tested.

We tested the reproducibility of the assay and the durability of stored cfDNA (**Figure S2C**) and obtained equivalent results with cfDNA extracted from plasma that had been stored at -80 for at least 4 years. We also tested longitudinal samples from a selection of patients and found that patients with stable disease had stable plasma cfDNA PVLs, whereas plasma cfDNA PVL tracked disease progression and was an early indicator of response to therapy (**Figure 3**).

## 4 Discussion

By isolating high quality cfDNA from peripheral blood samples, we showed for the first time that HTLV-1 infection is associated with increased concentrations of cfDNA in the blood plasma, and that proviral DNA is a component of blood plasma cfDNA in HTLV-1 carriers.

Although our estimates of the total amount of cfDNA recovered varied significantly according to the method that was used for quantification, groups with HTLV-1 associated diseases had systematically higher amounts of circulating cfDNA than uninfected controls, with the highest levels of cfDNA observed in the ATL-NR group. The cause of the increased total amount of cfDNA circulating in HTLV-1 carriers is not known, and could not be accounted for by cell death of infected cells alone.

HTLV-1 proviruses were detected in plasma cfDNA from >85% HTLV-1 carriers, and lack of detection of proviruses was associated with low PBMC gDNA PVLs. In our study, the PVL measured in plasma cfDNA was significantly lower than the PBMC PVL. This is expected, as (1) most plasma cfDNA is derived from haematopoietic lineage cells which include subsets that turnover more rapidly than lymphocytes (e.g. neutrophils), thus should contribute a greater fraction of DNA to the cfDNA pool; (2) other dying (nonhaematopoietic lineage) cells in the body also contribute to the plasma cfDNA pool; (3) cfDNA is highly fragmented, so PCR is likely to underestimate the absolute abundance of any sequence present.

The plasma cfDNA PVL correlated with PBMC gDNA PVL in the AC, HAM and ATL-R groups, showing that the plasma cfDNA PVL is proportional to the PBMC gDNA PVL in most HTLV-1 carriers, and allowing us to predict a person’s plasma cfDNA PVL from their PBMC gDNA PVL. In the ATL-NR group, a higher than expected plasma cfDNA PVL was observed relative to each patient’s PBMC gDNA PVL. Importantly, our data suggests that in the ATL-NR group a median of 10% of plasma cfDNA is derived from malignant cells. This fraction of tumour-derived DNA is sufficiently high to allow mutational profiling of ATL in cfDNA using standard sequencing protocols.

The potential clinical utility of our assay is underlined by our longitudinal data, which shows that though the half-life of blood plasma cfDNA is less than an hour, the plasma cfDNA PVL was highly reproducible in samples taken at an interval of >1 month in two HTLV-1 carriers with stable clinical statuses. In contrast, in one patient with evolving from chronic to acute ATL the plasma cfDNA PVL increased significantly, and in another patient with ATL who responded to therapy the plasma cfDNA PVL declined to undetectable levels, despite there being no significant change in the PVL of PBMCs.

This novel insight into the effect of HTLV-1 infection on the composition of cfDNA has potential applications in both research and clinical settings. Our approach could be used to address long standing research questions e.g. effect of drug therapy for HAM on the turnover HTLV-1 infected cells *in vivo*. Our data provides proof-of principle that assessment of HTLV-1 proviruses in cfDNA could be used clinically to monitor ATL patients during treatment, and in remission, to quantify residual disease and detect relapse early. Further studies are underway to test whether mutational profiling of ATL-driver mutations in cfDNA can offer clinically useful information about the genetic composition of lymphoma type ATL.

## Data availability statement

The original contributions presented in the study are included in the article/**Supplementary Material**. Further inquiries can be directed to the corresponding author.

## Ethics statement

Blood donors attended the National Centre for Human Retrovirology (Imperial College Healthcare NHS Trust, St Mary’s Hospital, London). Research was conducted under the governance of the Communicable Diseases Research Group Tissue Bank, approved by the UK National Research Ethics Service (09/H0606/106, 15/SC/0089, 20/SC/0226). The patients/participants provided their written informed consent to participate in this study.

## Author contributions

AR designed the study, TJ and AR performed the experimental work and data analysis, JH recruited the patients and LC and GT characterised the patients. All authors contributed to the article and approved the submitted version.
