# Supplementary material for: Detection of HTLV-1 proviral DNA in cell-free DNA: Potential for non-invasive monitoring of Adult T cell leukaemia/lymphoma using liquid biopsy?
Source: Front Immunol. 2023 Apr 11;14:1150285. doi: 10.3389/fimmu.2023.1150285 (PMC10126272; doi:10.3389/fimmu.2023.1150285)
Supplement: Supplementary file 1 [file Presentation_1.pptx]

## Slide 1
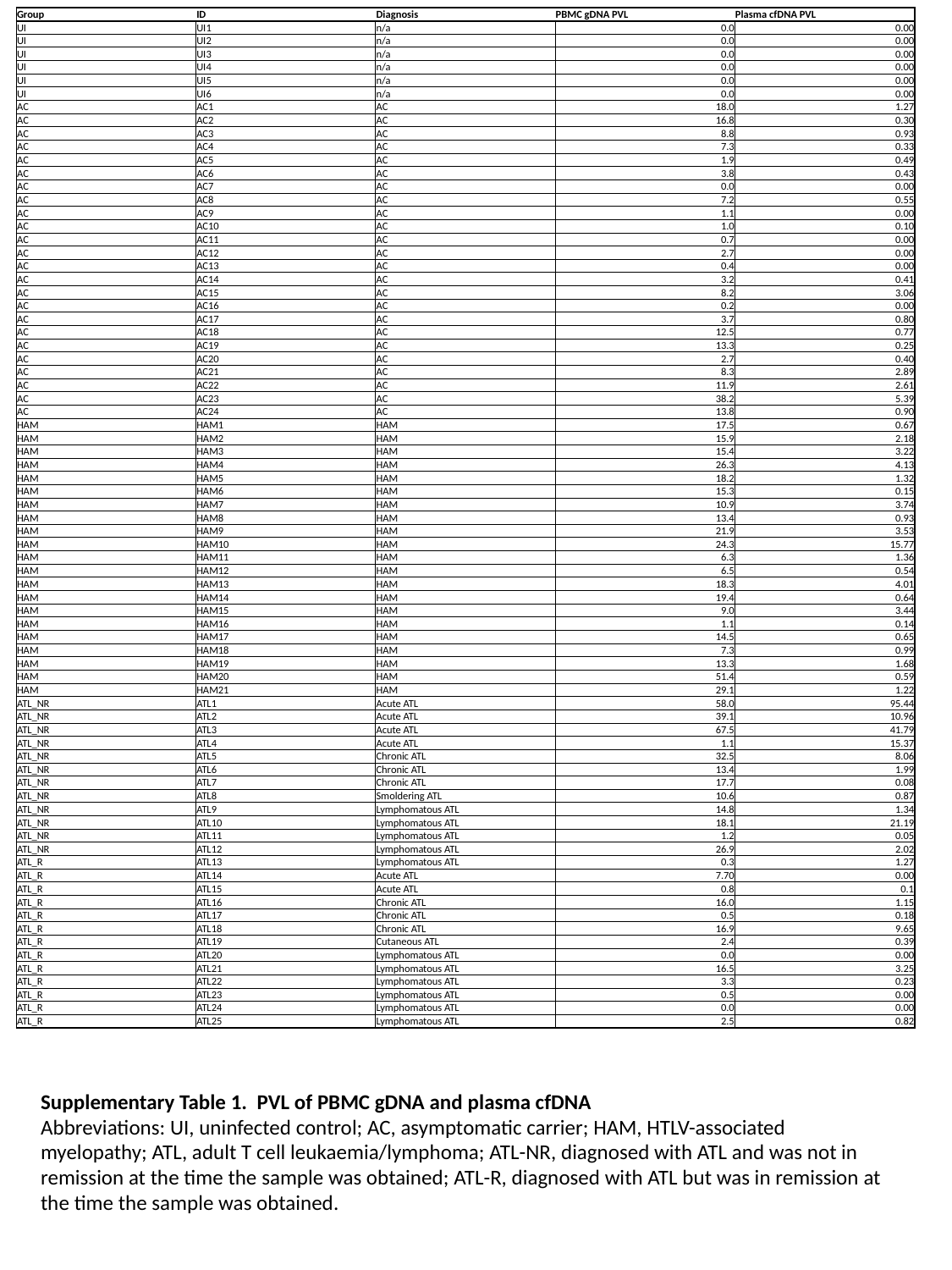

| Group | ID | Diagnosis | PBMC gDNA PVL | Plasma cfDNA PVL |
| --- | --- | --- | --- | --- |
| UI | UI1 | n/a | 0.0 | 0.00 |
| UI | UI2 | n/a | 0.0 | 0.00 |
| UI | UI3 | n/a | 0.0 | 0.00 |
| UI | UI4 | n/a | 0.0 | 0.00 |
| UI | UI5 | n/a | 0.0 | 0.00 |
| UI | UI6 | n/a | 0.0 | 0.00 |
| AC | AC1 | AC | 18.0 | 1.27 |
| AC | AC2 | AC | 16.8 | 0.30 |
| AC | AC3 | AC | 8.8 | 0.93 |
| AC | AC4 | AC | 7.3 | 0.33 |
| AC | AC5 | AC | 1.9 | 0.49 |
| AC | AC6 | AC | 3.8 | 0.43 |
| AC | AC7 | AC | 0.0 | 0.00 |
| AC | AC8 | AC | 7.2 | 0.55 |
| AC | AC9 | AC | 1.1 | 0.00 |
| AC | AC10 | AC | 1.0 | 0.10 |
| AC | AC11 | AC | 0.7 | 0.00 |
| AC | AC12 | AC | 2.7 | 0.00 |
| AC | AC13 | AC | 0.4 | 0.00 |
| AC | AC14 | AC | 3.2 | 0.41 |
| AC | AC15 | AC | 8.2 | 3.06 |
| AC | AC16 | AC | 0.2 | 0.00 |
| AC | AC17 | AC | 3.7 | 0.80 |
| AC | AC18 | AC | 12.5 | 0.77 |
| AC | AC19 | AC | 13.3 | 0.25 |
| AC | AC20 | AC | 2.7 | 0.40 |
| AC | AC21 | AC | 8.3 | 2.89 |
| AC | AC22 | AC | 11.9 | 2.61 |
| AC | AC23 | AC | 38.2 | 5.39 |
| AC | AC24 | AC | 13.8 | 0.90 |
| HAM | HAM1 | HAM | 17.5 | 0.67 |
| HAM | HAM2 | HAM | 15.9 | 2.18 |
| HAM | HAM3 | HAM | 15.4 | 3.22 |
| HAM | HAM4 | HAM | 26.3 | 4.13 |
| HAM | HAM5 | HAM | 18.2 | 1.32 |
| HAM | HAM6 | HAM | 15.3 | 0.15 |
| HAM | HAM7 | HAM | 10.9 | 3.74 |
| HAM | HAM8 | HAM | 13.4 | 0.93 |
| HAM | HAM9 | HAM | 21.9 | 3.53 |
| HAM | HAM10 | HAM | 24.3 | 15.77 |
| HAM | HAM11 | HAM | 6.3 | 1.36 |
| HAM | HAM12 | HAM | 6.5 | 0.54 |
| HAM | HAM13 | HAM | 18.3 | 4.01 |
| HAM | HAM14 | HAM | 19.4 | 0.64 |
| HAM | HAM15 | HAM | 9.0 | 3.44 |
| HAM | HAM16 | HAM | 1.1 | 0.14 |
| HAM | HAM17 | HAM | 14.5 | 0.65 |
| HAM | HAM18 | HAM | 7.3 | 0.99 |
| HAM | HAM19 | HAM | 13.3 | 1.68 |
| HAM | HAM20 | HAM | 51.4 | 0.59 |
| HAM | HAM21 | HAM | 29.1 | 1.22 |
| ATL\_NR | ATL1 | Acute ATL | 58.0 | 95.44 |
| ATL\_NR | ATL2 | Acute ATL | 39.1 | 10.96 |
| ATL\_NR | ATL3 | Acute ATL | 67.5 | 41.79 |
| ATL\_NR | ATL4 | Acute ATL | 1.1 | 15.37 |
| ATL\_NR | ATL5 | Chronic ATL | 32.5 | 8.06 |
| ATL\_NR | ATL6 | Chronic ATL | 13.4 | 1.99 |
| ATL\_NR | ATL7 | Chronic ATL | 17.7 | 0.08 |
| ATL\_NR | ATL8 | Smoldering ATL | 10.6 | 0.87 |
| ATL\_NR | ATL9 | Lymphomatous ATL | 14.8 | 1.34 |
| ATL\_NR | ATL10 | Lymphomatous ATL | 18.1 | 21.19 |
| ATL\_NR | ATL11 | Lymphomatous ATL | 1.2 | 0.05 |
| ATL\_NR | ATL12 | Lymphomatous ATL | 26.9 | 2.02 |
| ATL\_R | ATL13 | Lymphomatous ATL | 0.3 | 1.27 |
| ATL\_R | ATL14 | Acute ATL | 7.70 | 0.00 |
| ATL\_R | ATL15 | Acute ATL | 0.8 | 0.1 |
| ATL\_R | ATL16 | Chronic ATL | 16.0 | 1.15 |
| ATL\_R | ATL17 | Chronic ATL | 0.5 | 0.18 |
| ATL\_R | ATL18 | Chronic ATL | 16.9 | 9.65 |
| ATL\_R | ATL19 | Cutaneous ATL | 2.4 | 0.39 |
| ATL\_R | ATL20 | Lymphomatous ATL | 0.0 | 0.00 |
| ATL\_R | ATL21 | Lymphomatous ATL | 16.5 | 3.25 |
| ATL\_R | ATL22 | Lymphomatous ATL | 3.3 | 0.23 |
| ATL\_R | ATL23 | Lymphomatous ATL | 0.5 | 0.00 |
| ATL\_R | ATL24 | Lymphomatous ATL | 0.0 | 0.00 |
| ATL\_R | ATL25 | Lymphomatous ATL | 2.5 | 0.82 |
Supplementary Table 1. PVL of PBMC gDNA and plasma cfDNA
Abbreviations: UI, uninfected control; AC, asymptomatic carrier; HAM, HTLV-associated myelopathy; ATL, adult T cell leukaemia/lymphoma; ATL-NR, diagnosed with ATL and was not in remission at the time the sample was obtained; ATL-R, diagnosed with ATL but was in remission at the time the sample was obtained.

## Slide 2
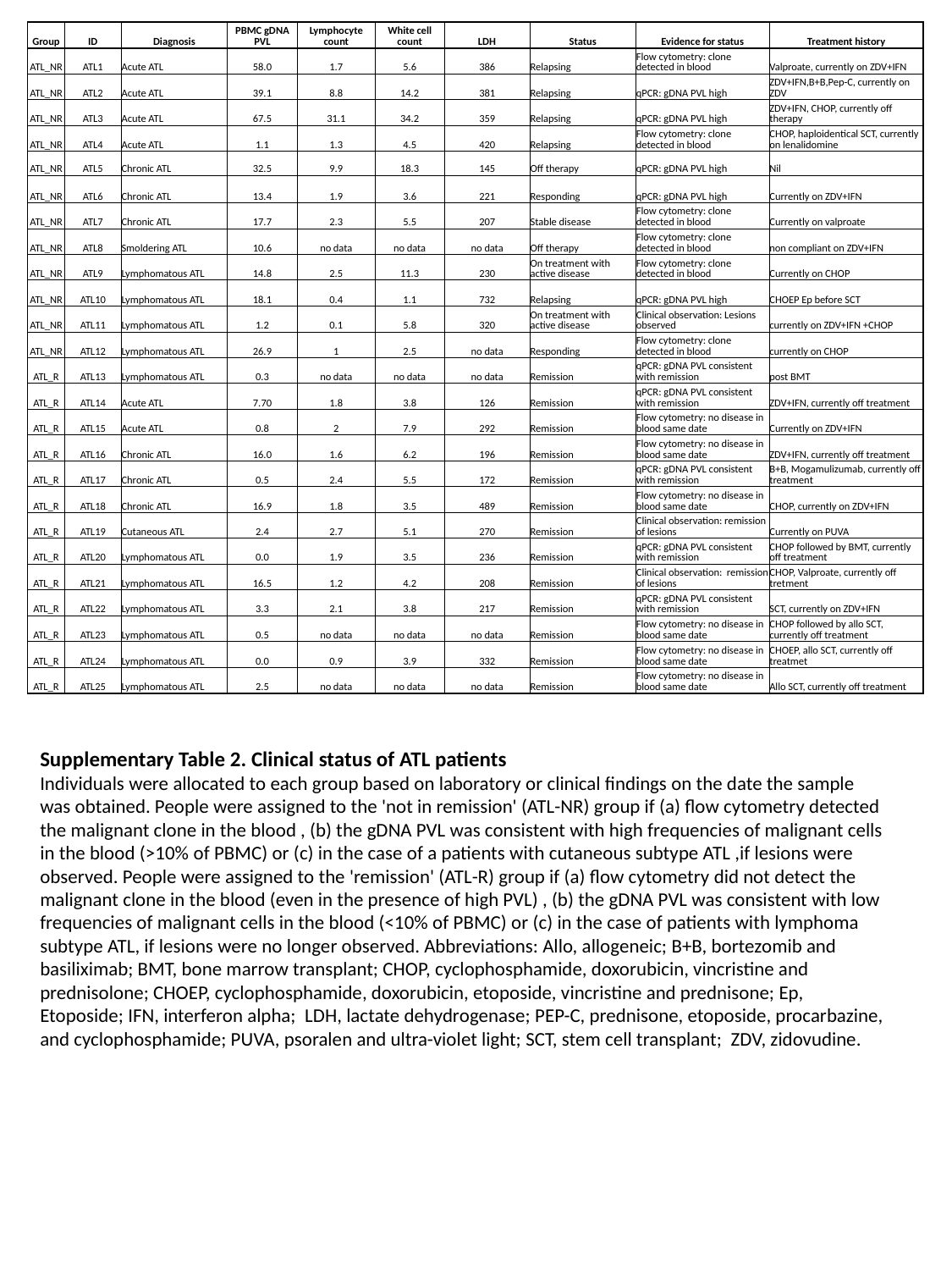

| Group | ID | Diagnosis | PBMC gDNA PVL | Lymphocyte count | White cell count | LDH | Status | Evidence for status | Treatment history |
| --- | --- | --- | --- | --- | --- | --- | --- | --- | --- |
| ATL\_NR | ATL1 | Acute ATL | 58.0 | 1.7 | 5.6 | 386 | Relapsing | Flow cytometry: clone detected in blood | Valproate, currently on ZDV+IFN |
| ATL\_NR | ATL2 | Acute ATL | 39.1 | 8.8 | 14.2 | 381 | Relapsing | qPCR: gDNA PVL high | ZDV+IFN,B+B,Pep-C, currently on ZDV |
| ATL\_NR | ATL3 | Acute ATL | 67.5 | 31.1 | 34.2 | 359 | Relapsing | qPCR: gDNA PVL high | ZDV+IFN, CHOP, currently off therapy |
| ATL\_NR | ATL4 | Acute ATL | 1.1 | 1.3 | 4.5 | 420 | Relapsing | Flow cytometry: clone detected in blood | CHOP, haploidentical SCT, currently on lenalidomine |
| ATL\_NR | ATL5 | Chronic ATL | 32.5 | 9.9 | 18.3 | 145 | Off therapy | qPCR: gDNA PVL high | Nil |
| ATL\_NR | ATL6 | Chronic ATL | 13.4 | 1.9 | 3.6 | 221 | Responding | qPCR: gDNA PVL high | Currently on ZDV+IFN |
| ATL\_NR | ATL7 | Chronic ATL | 17.7 | 2.3 | 5.5 | 207 | Stable disease | Flow cytometry: clone detected in blood | Currently on valproate |
| ATL\_NR | ATL8 | Smoldering ATL | 10.6 | no data | no data | no data | Off therapy | Flow cytometry: clone detected in blood | non compliant on ZDV+IFN |
| ATL\_NR | ATL9 | Lymphomatous ATL | 14.8 | 2.5 | 11.3 | 230 | On treatment with active disease | Flow cytometry: clone detected in blood | Currently on CHOP |
| ATL\_NR | ATL10 | Lymphomatous ATL | 18.1 | 0.4 | 1.1 | 732 | Relapsing | qPCR: gDNA PVL high | CHOEP Ep before SCT |
| ATL\_NR | ATL11 | Lymphomatous ATL | 1.2 | 0.1 | 5.8 | 320 | On treatment with active disease | Clinical observation: Lesions observed | currently on ZDV+IFN +CHOP |
| ATL\_NR | ATL12 | Lymphomatous ATL | 26.9 | 1 | 2.5 | no data | Responding | Flow cytometry: clone detected in blood | currently on CHOP |
| ATL\_R | ATL13 | Lymphomatous ATL | 0.3 | no data | no data | no data | Remission | qPCR: gDNA PVL consistent with remission | post BMT |
| ATL\_R | ATL14 | Acute ATL | 7.70 | 1.8 | 3.8 | 126 | Remission | qPCR: gDNA PVL consistent with remission | ZDV+IFN, currently off treatment |
| ATL\_R | ATL15 | Acute ATL | 0.8 | 2 | 7.9 | 292 | Remission | Flow cytometry: no disease in blood same date | Currently on ZDV+IFN |
| ATL\_R | ATL16 | Chronic ATL | 16.0 | 1.6 | 6.2 | 196 | Remission | Flow cytometry: no disease in blood same date | ZDV+IFN, currently off treatment |
| ATL\_R | ATL17 | Chronic ATL | 0.5 | 2.4 | 5.5 | 172 | Remission | qPCR: gDNA PVL consistent with remission | B+B, Mogamulizumab, currently off treatment |
| ATL\_R | ATL18 | Chronic ATL | 16.9 | 1.8 | 3.5 | 489 | Remission | Flow cytometry: no disease in blood same date | CHOP, currently on ZDV+IFN |
| ATL\_R | ATL19 | Cutaneous ATL | 2.4 | 2.7 | 5.1 | 270 | Remission | Clinical observation: remission of lesions | Currently on PUVA |
| ATL\_R | ATL20 | Lymphomatous ATL | 0.0 | 1.9 | 3.5 | 236 | Remission | qPCR: gDNA PVL consistent with remission | CHOP followed by BMT, currently off treatment |
| ATL\_R | ATL21 | Lymphomatous ATL | 16.5 | 1.2 | 4.2 | 208 | Remission | Clinical observation: remission of lesions | CHOP, Valproate, currently off tretment |
| ATL\_R | ATL22 | Lymphomatous ATL | 3.3 | 2.1 | 3.8 | 217 | Remission | qPCR: gDNA PVL consistent with remission | SCT, currently on ZDV+IFN |
| ATL\_R | ATL23 | Lymphomatous ATL | 0.5 | no data | no data | no data | Remission | Flow cytometry: no disease in blood same date | CHOP followed by allo SCT, currently off treatment |
| ATL\_R | ATL24 | Lymphomatous ATL | 0.0 | 0.9 | 3.9 | 332 | Remission | Flow cytometry: no disease in blood same date | CHOEP, allo SCT, currently off treatmet |
| ATL\_R | ATL25 | Lymphomatous ATL | 2.5 | no data | no data | no data | Remission | Flow cytometry: no disease in blood same date | Allo SCT, currently off treatment |
Supplementary Table 2. Clinical status of ATL patients
Individuals were allocated to each group based on laboratory or clinical findings on the date the sample was obtained. People were assigned to the 'not in remission' (ATL-NR) group if (a) flow cytometry detected the malignant clone in the blood , (b) the gDNA PVL was consistent with high frequencies of malignant cells in the blood (>10% of PBMC) or (c) in the case of a patients with cutaneous subtype ATL ,if lesions were observed. People were assigned to the 'remission' (ATL-R) group if (a) flow cytometry did not detect the malignant clone in the blood (even in the presence of high PVL) , (b) the gDNA PVL was consistent with low frequencies of malignant cells in the blood (<10% of PBMC) or (c) in the case of patients with lymphoma subtype ATL, if lesions were no longer observed. Abbreviations: Allo, allogeneic; B+B, bortezomib and basiliximab; BMT, bone marrow transplant; CHOP, cyclophosphamide, doxorubicin, vincristine and prednisolone; CHOEP, cyclophosphamide, doxorubicin, etoposide, vincristine and prednisone; Ep, Etoposide; IFN, interferon alpha; LDH, lactate dehydrogenase; PEP-C, prednisone, etoposide, procarbazine, and cyclophosphamide; PUVA, psoralen and ultra-violet light; SCT, stem cell transplant; ZDV, zidovudine.

## Slide 3
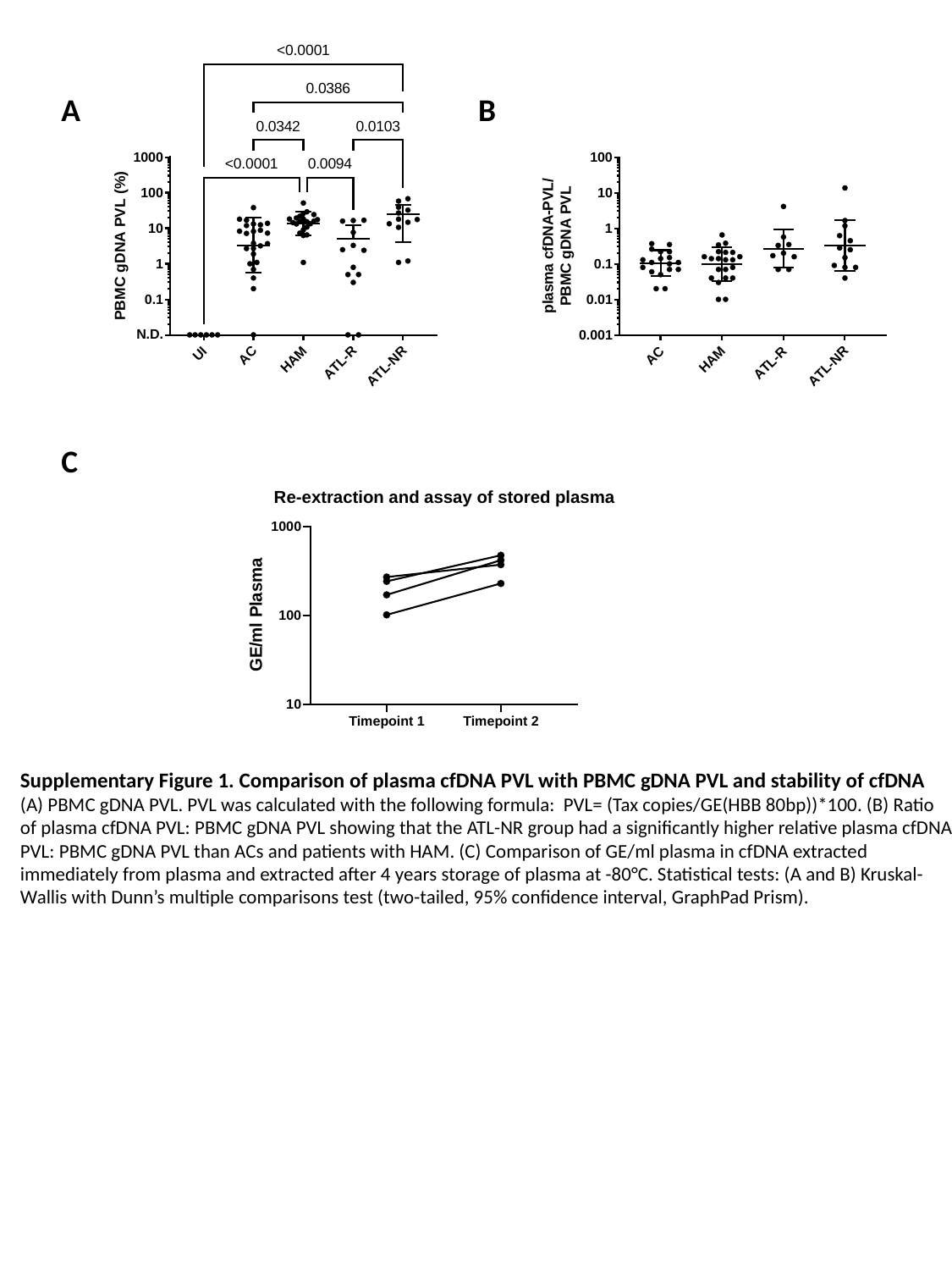

A
B
C
Supplementary Figure 1. Comparison of plasma cfDNA PVL with PBMC gDNA PVL and stability of cfDNA
(A) PBMC gDNA PVL. PVL was calculated with the following formula: PVL= (Tax copies/GE(HBB 80bp))*100. (B) Ratio of plasma cfDNA PVL: PBMC gDNA PVL showing that the ATL-NR group had a significantly higher relative plasma cfDNA PVL: PBMC gDNA PVL than ACs and patients with HAM. (C) Comparison of GE/ml plasma in cfDNA extracted immediately from plasma and extracted after 4 years storage of plasma at -80°C. Statistical tests: (A and B) Kruskal-Wallis with Dunn’s multiple comparisons test (two-tailed, 95% confidence interval, GraphPad Prism).
